# Oral lichen planus in children: A systematic review

**DOI:** 10.4317/medoral.25938

**Published:** 2024-01-30

**Authors:** Francesca Spirito, Mario Dioguardi, Vito Carlo Alberto Caponio, Mariateresa Ambrosino, Eleonora Lo Muzio, Lorenzo Lo Muzio

**Affiliations:** 1 Department of Clinical and Experimental Medicine, University of Foggia, Foggia, Italy; 2 School of Orthodontics, University of Ferrara, Ferrara, Italy

## Abstract

**Background:**

Oral Lichen Planus is a common chronic inflammatory disease of the oral mucosa. The prevalence in adults ranges between 0.5% and 2%, while in children is reported to be about 0,03%. Clinical features of Oral Lichen Planus could be variable in both adults and children, ranging from painless white hyperkeratotic lesions to painful erythematous atrophic ones.

Actually, there are no systematic reviews in the literature on OLP in children, whereby this paper aims to summarize all the pathophysiological aspects and identify all cases described in the literature of Oral Lichen Planus in children, reporting their clinical characteristics.

**Material and Methods:**

A systematic review of the literature was performed in online databases including PubMed, Scopus, Web of Science, Science Direct, EMBASE. In addition, in order to identify reports not otherwise identifiable, an analysis of the gray literature was performed on google scholar and in Open Gray.

**Results:**

By literature analysis, it emerged that most cases were reported from India. The mean age at time of diagnosis of the disease was 11 years, ranging from 3 to 17 years. The most frequent pattern was the reticular pattern followed by plaque-like, erosive, atrophic, sclerosus, and bullous. The buccal mucosa was the most involved oral site, followed by the tongue, lips and gingiva.

**Conclusions:**

Although Oral Lichen Planus in children is rare, it may cause oral discomfort and need to be differentiated from other oral white lesions and/or chronic ulcers.

** Key words:**Oral lichen planus, childhood, lichen ruber planus, paediatric lichen planus.

## Introduction

Oral Lichen Planus (OLP) is a common chronic inflammatory disease of oral mucosa (1,2). The prevalence in adults ranges between 0.5% and 2% (1), with the highest incidence in the third to sixth decade of life (3), and a male-to-female ratio ranging from 1:2 (4) to 1:3 (2). Disease prevalence in children is actually low: it is reported to be about 0.03% (5); most of the cases are from Asian countries, especially India (6,7).

The aetiology OLP is still unclear. It seems that an autoimmune response (8) involving antigen-presenting cells and regulatory T-lymphocytes, probably triggered by keratinocytes, plays a key role in the development of the disease (9). Histopathologically, OLP shows typical degeneration of the basal cell layer with apoptosis of the keratinocytes, a dense band-like lymphocytic infiltrate at the interface between the epithelium and the connective tissue, focal areas of hyperkeratinized epithelium, and, occasionally, atrophic areas (10). A number of auto-immune diseases, including lupus erythematous, pemphigus, rheumatoid arthritis, and Sjogren’s syndrome, have been reported in association to OLP (5).

For paediatric lichen planus, possible associations with hepatitis B virus (HBV) or hepatitis B vaccination have been described (11). Moreover, genetic factors, lifestyle, and emotional stress might be contributing factors in OLP pathogenesis (12,13). In particular, in patients with paediatric lichen planus a higher incidence of positive family history for the disease is reported (14); on the other hand, genetic linkage studies reported an association between familial lichen planus and HLA B, HLA DR1 and DR10 (15).

Clinical features of OLP could be variable in both adults and children, ranging from painless white hyperkeratotic lesions (frequently symmetrical with papules, plaques, or Wickham striae) to painful erythematous atrophic ones (sometimes with blisters, erosions and ulcerations) (16). Cutaneous lichen planus is often self-limiting, whereas OLP tends to be chronic: spontaneous remission has been rarely reported in OLP (2); in general, OLP prognosis in children seems to be more favourable (12) .

The differential diagnosis is strictly related to clinical features of OLP and includes oral candidiasis, morsicatio buccarum, lichenoid drug reaction, leucoplakia, lupus erythematous, graft versus host disease (GVHD), and others.

A recent review of the literature including case series was carried out by Shikha G. *et al*. (17). The analysis of the literature, even if not carried out in a systematic way, allowed to identify 28 manuscripts in the scientific literature for a total of 42 cases of OLP. From the results it emerges that the first case was reported in 1992 while the patient's age range was 3 to 14 years with a mean age of 9.4 years (males 20 and females 22).

At present there are no systematic reviews in the literature on OLP in children, while reviews on Lichen planus (LP) in pediatric age have been performed; Specifically, a recent systematic review has identified all cases described in the literature of lichen Planus pigmentosum which would represent 2.8% of Children with LP without referring to oral manifestations(18-20), and other reviews have focused on the role of vaccinations for hepatitis B in association with the onset of LP in pediatric age (19,20).

Given the importance of intercepting neoformations and oral precancerous diseases in children, it is essential to understand the real impact of OLP cases in children and its clinical course in terms of follow-up.

With this Review, therefore, we set ourselves the aim of summarizing all the pathophysiological aspects and identifying all cases described in the literature of OLP in children, reporting their clinical characteristics.

- List of abbreviations.

Oral Lichen Planus (OLP); Hepatitis B virus (HBV); Graft Versus Host Disease (GVHD); Lichen Planus (LP).

## Material and Methods

- Protocol and registration

The planning of the systematic review was performed according to the recommendations of the Cochrane Handbook. The review was written following the indications of the PRISMA (Preferred Reporting Items for Systematic Reviews and Meta-Analysis) (21), the protocol was registered before to proceed with the drafting of the manuscript on the on the International Platform of Registered Systematic Review and Meta-analysis Protocols (INPLASY), with registration number INPLASY202290106 and DOI number 10.37766/inplasy2022.9.0106

- Eligibility criteria

All clinical studies (retrospective, prospective and randomized trials) case series and case reports reporting cases of OLP in children were considered potentially admissible, while systematic reviews on OLP were only consulted in order not to repeat the review work, and as a source of bibliographic references. The review question asked is to identify all clinical cases described in the literature of OLP in children, reporting their clinical and pathophysiological characteristics.

The selection of the studies was based on the following inclusion criteria; case series or case reports; Age<18 years old at the time of diagnosis; clinical and histological diagnosis of OLP; An accurate description of the oral sites and clinical features. The exclusion criteria were: Graft Versus Host Disease lichenoid lesions; No histology; Oral lichenoid drug reaction; No data about clinical form and/or oral sites involved.

No language restrictions were applied, therefore all studies and reports were taken into consideration, even in a language other than English, for which at least one English translation of the abstract was available.

- Sources of information, research and selection

Before proceeding with the research and selection phase of the studies, the 3 researchers assigned to carry out the research and selection were identified, the first 2 with the task of identifying the articles and the 3 to decide in doubtful situations.

The research and selection phase of the articles therefore took place in the following phases:

1) Choice of inclusion and exclusion criteria, databases and keywords to be used and the time period in which to conduct the research;

2) Search and selection of records on databases carried out independently;

3) Removal of overlaps manually and using reference management software such as EndNote 8.0;

4) Choice of studies to be included (carried out independently by the 2 researchers);

5) Comparison of the included studies and resolution of any conflicts between the 2 reviewers with the help, if necessary, of a 3rd reviewer.

A systematic review of literature was performed in the online databases including PubMed, Scopus, Web of Science, Science Direct, EMBASE on 31 May 2022, in addition, an analysis of the gray literature was performed on google scholar and in Open Gray (DANS EASY Archive) in order to identify reports not otherwise identifiable.

All the relevant papers and reports published in English from January 1966 through may 2022 were extracted. Several combinations of keywords were used in the following orders to conduct the search strategy: 1) “Lichen Planus’’ OR “Oral Lichen Planus’’ AND 2) “Children” OR ‘’Child’’ OR “Childhood” OR “Paediatric” OR ‘’Pediatric’’.

Specifically we report from PubMed, in detail all the combinations of keywords used by the portal:

Search: Lichen Planus AND (Children OR pediatric) Sort by: Most Recent

("lichen planus"[MeSH Terms] OR ("lichen"[All Fields] AND "planus"[All Fields]) OR "lichen planus"[All Fields]) AND ("child"[MeSH Terms] OR "child"[All Fields] OR "children"[All Fields] OR "child s"[All Fields] OR "children s"[All Fields] OR "childrens"[All Fields] OR "childs"[All Fields] OR ("paediatrics"[All Fields] OR "pediatrics"[MeSH Terms] OR "pediatrics"[All Fields] OR "paediatric"[All Fields] OR "pediatric"[All Fields]))

Translations; Lichen Planus: "lichen planus"[MeSH Terms] OR ("lichen"[All Fields] AND "planus"[All Fields]) OR "lichen planus"[All Fields]

Children: "child"[MeSH Terms] OR "child"[All Fields] OR "children"[All Fields] OR "child's"[All Fields] OR "children's"[All Fields] OR "childrens"[All Fields] OR "childs"[All Fields]

pediatric: "paediatrics"[All Fields] OR "pediatrics"[MeSH Terms] OR "pediatrics"[All Fields] OR "paediatric"[All Fields] OR "pediatric"[All Fields]

Two independent investigators retrieved the studies that were the most relevant by titles and abstracts. Subsequently, the full text of the retrieved papers was reviewed, and the most relevant papers were chosen according to the eligibility criteria. Duplicate results were removed using the EndNote 8 software, the overlaps of studies that could not be uploaded to EndNote were manually removed after the screening phase.

In addition, an update of the research on bibliographic sources was carried out on 01 September 2023.

- Data collection process, Data characteristics.

The data to be extracted from the included articles were decided in advance by the 3 researchers and concerned: the first Author, the country where the study was conducted, the year of publication, the patient's age, sex, lesion morphology, the presence of pain, the cutaneous involvement of the lesions, the type of treatment and the follow-up. The data were reported by the 2 researchers in 2 different Tables and subsequently compared and checked by the 3 researchers in order to minimize the error in reporting the data in a single Table.

Then, the relevant data were extracted and organized in Table 1.

- Risk of Bias.

The risk of bias was evaluated using a tool relating to case reports. The tool used for case reports is the JBI critical appraisal checklist for case reports (22). The evaluation was performed by a researcher after the data extraction and inclusion phase of the studies.

## Results

- Selection of studies

**Figure 1:**
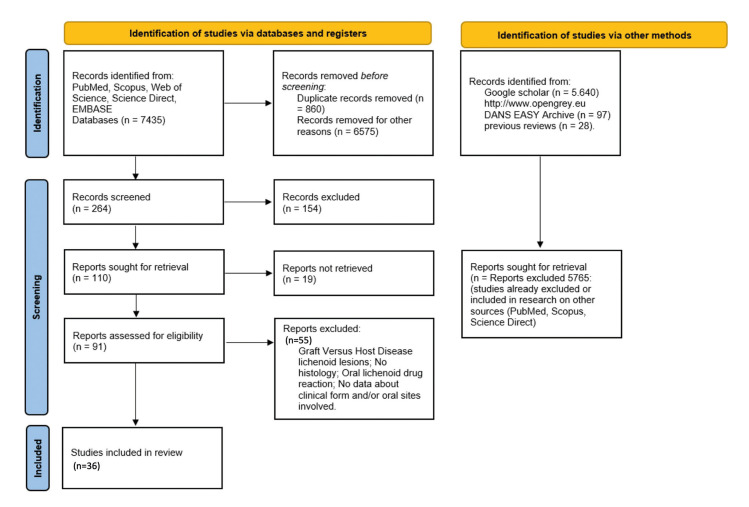
Flow diagram for the selection process of identified articles.

The analysis of literature between 1966 and 2023 allowed the selection of 7447 papers (Science Direct, SCOPUS, PubMed, EBSCO, Web of Science). After the application of the inclusion/exclusion criteria, only 36 papers (5,6,12,16,17,23-53) were included in the present systematic review (Fig. 1) with 65 cases (Table 1).

Furthermore, the analysis of the gray literature (http://www.opengrey.eu DANS EASY Archive and Google Scholar) and previous systematic reviews did not allow the identification of further OLP cases to be included in the qualitative assessment. The entire procedure of identification, selection and inclusion of the studies has been indicated in the flowchart of Fig. 1.

The risk of bias was assessed as accepTable for all included studies (Table 2)

- Data characteristics

First of all, if we consider the geographical location, the most interested country is India (23/65, 35.4%), followed by Italy (17/65, 26.1%), UK (7/65, 10.7%), USA (3/65, 4.6%), France (3/65, 4.6%) and Brazil (3/65, 4.6%).

**Figure 2:**
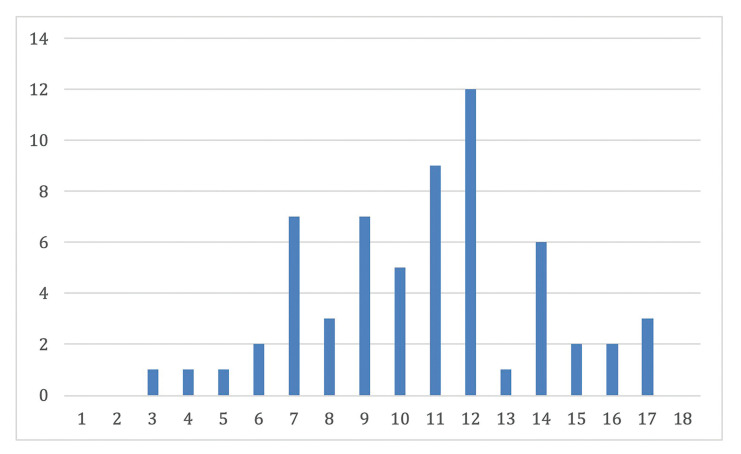
Case distribution according to age.

The mean age at time of diagnosis of the disease was 11 years, ranging from 3 to 17 years (Fig. 2). The most frequent pattern was the reticular pattern (28/65, 43%), followed by plaque-like (15/65, 23%), erosive/ulcerative (12/65 18.4%), papular (10/65, 15.3%), atrophic (8/65, 12.3%), sclerosus (3/65, 4.6%) and bullous (1/65, 1.5%) patterns. The buccal mucosa was the most commonly involved oral site (38/65, 58.4%), followed by tongue (27/65, 41.5%), lips (8/65, 12.3%) and gingiva (9/65, 13.8%). 32/65 patients (59.2%) were symptomatic. 12/65 (18.4%) patients showed skin involvement. 33 patients were male (50.7%) and 32 were female (49.2%). The treatment strategy are described in Table 1.

**Table 1:**
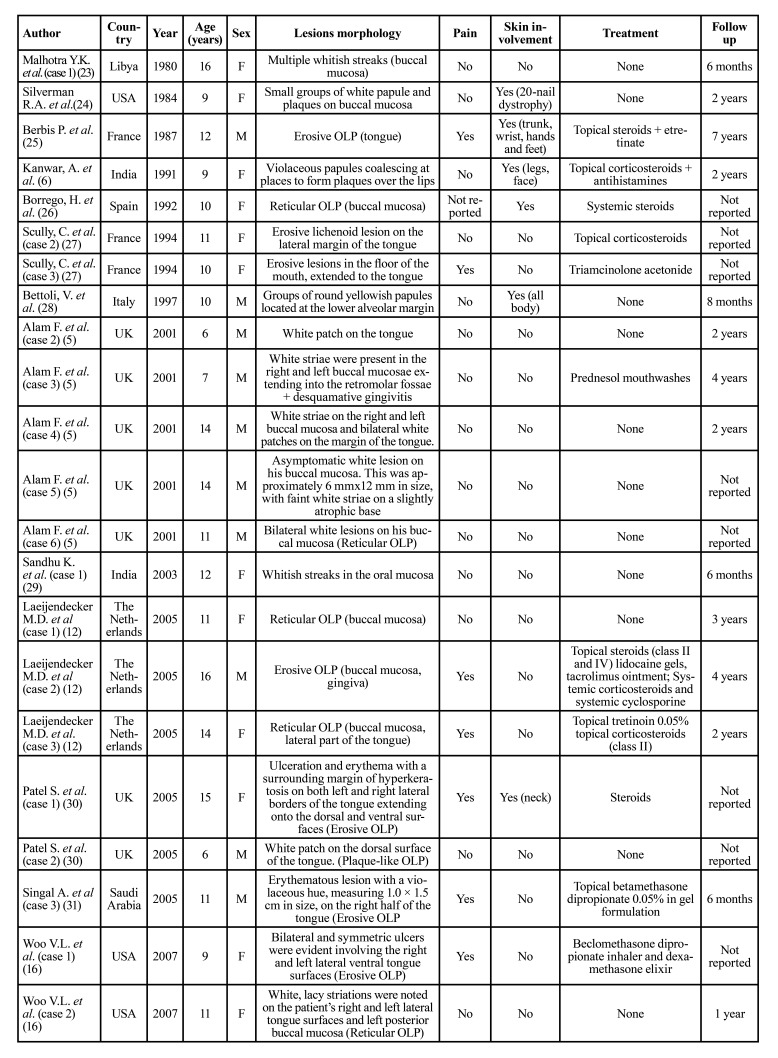
Studies of childhood LP in literature from 1980 to 2023.

**Table 1 cont.**
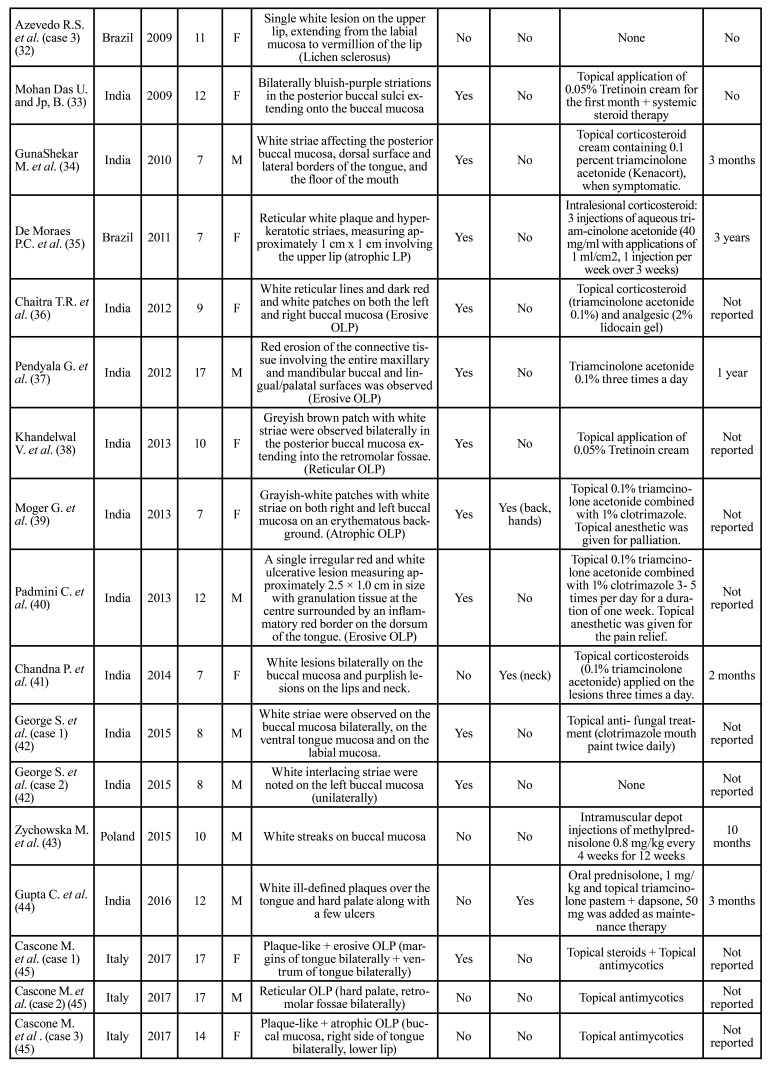
Studies of childhood LP in literature from 1980 to 2023.

**Table 1 cont.**
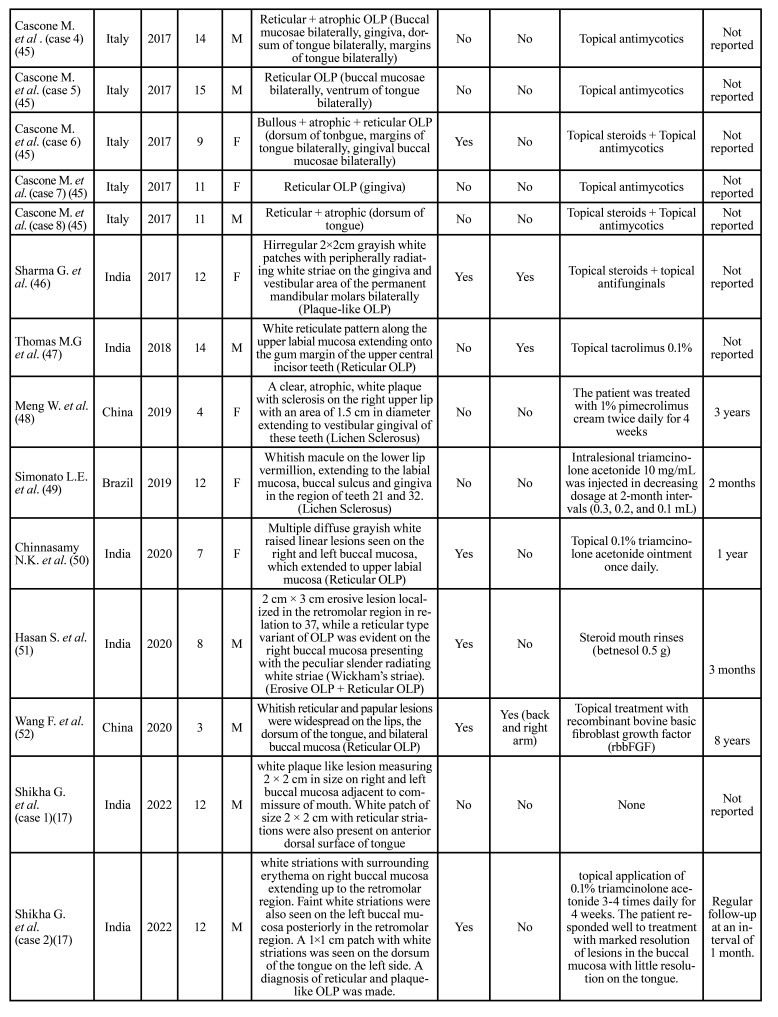
Studies of childhood LP in literature from 1980 to 2023.

**Table 1 cont.**
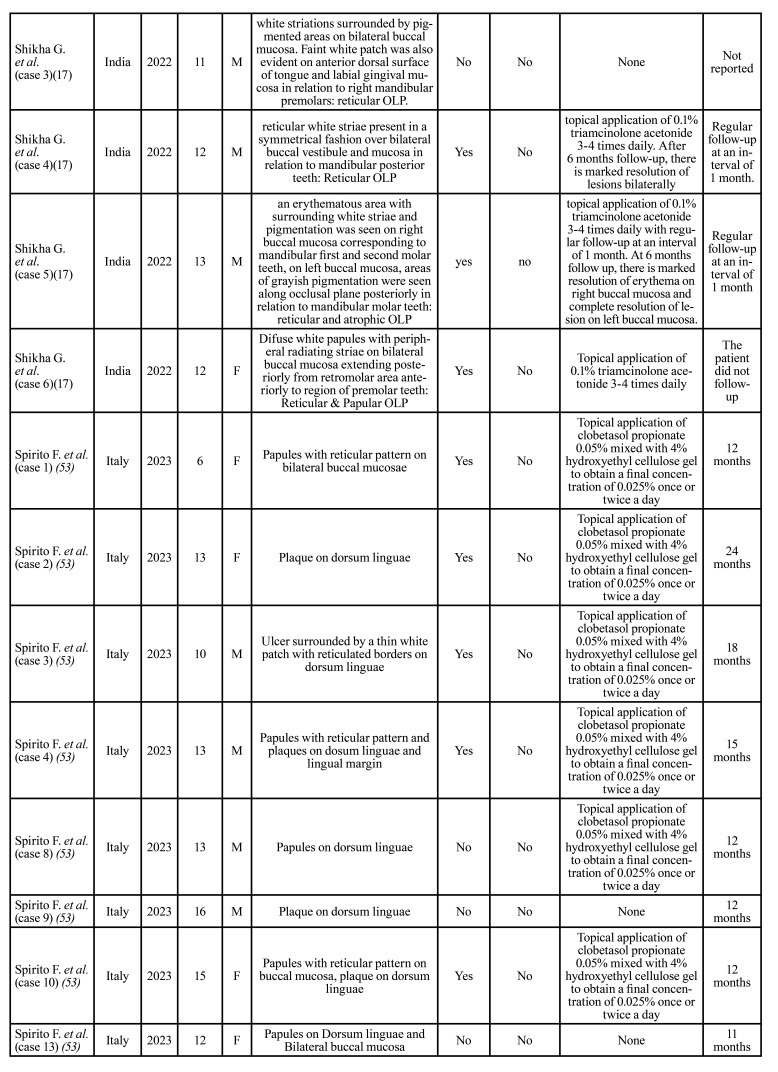
Studies of childhood LP in literature from 1980 to 2023.

**Table 2:**
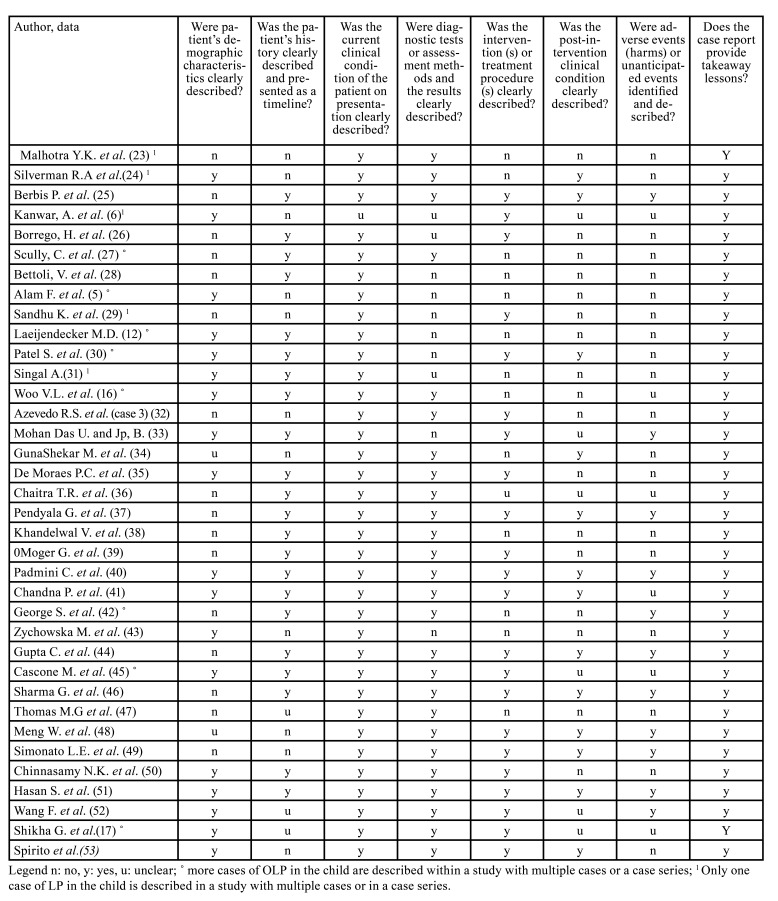
Risk of bias: JBI critical appraisal checklist for case reports.

## Discussion

This systematic literature review was reported following the PRISMA guidelines, and at the end of the selection process we included in our review 36 manuscripts including 22 case reports, the other manuscripts were reported as case series on Oral Lichen with pediatric cases or described family cases, with a total of 65 pediatric OLP cases.

Although OLP in children was initially described in 1920s, just few cases have been so far reported (51). In most of the studies mucocutaneous lichen planus has been addressed, while oral involvement has been often described in association with predisposing conditions, such as hepatitis and vaccination (54). In Table 1, 65 cases of oral childhood lichen planus published are detailed.

The most interested country was India. The mean age at time of diagnosis of the disease was 11 years, ranging from 3 to 17 years (Fig. 2). The most frequent patterns were the reticular and plaque-like patterns. The buccal mucosa was the most involved oral site, followed by tongue. 33 patients were male (50.7%) and 32 were female (49.2%).

It has been suggested that childhood LP often shows atypical clinical features; this seems to be confirmed also in our series. In fact, four of the seven patients here presented had lesions confined to the tongue; this is uncommon in adults, in whom most of the times patients had multiple oral sites of involvement, whereas single sites of involvement are uncommon (4). On the other hand, we found that OLPc histological features are not different from OLP in adults.

OLPc treatment outcomes seems to be more favourable than adulthood OLP (40). To date, neither trials, nor consensus about standardized therapy for OLPc exists; so many different treatment modalities have been reported in literature (ranging from local corticosteroids to immunosuppressive drugs) (3,12). In a recent case series of 13 OLPs in the child described by our research group, when a treatment was needed, a local therapy based on low-dose corticosteroids (clobetasol propionate 0.05% mixed with 4% hydroxyethyl cellulose gel to obtain a final concentration of 0.025% once or twice a day), was successful in obtaining a sTable remission of symptoms (53).

A limitation to this systematic review of the literature is the inclusion of studies and reports that describe only case reports or case series, in fact the absence in the literature of observational studies that clearly identify and with precise clinical stratification, the real incidence of Pediatric OLP.

OLPc is an extremely uncommon occurrence. Majority of the childhood OLP cases are not reported due to misdiagnosis by the physicians (51). Any mucosal lesion in children should be referred to the specialist for an early and precise diagnosis and treatment protocol (51). General OLPc usually has a much fairer prognosis and responds well with therapy. In conclusion, though lichen planus in children is uncommon and oral mucosal involvement quite rare, clinicians should be aware of its existence and management and its differential diagnosis in children presenting oral white lesions and/or chronic ulcers.
